# Electrical stimulation of the endopiriform nucleus attenuates epilepsy in rats by network modulation

**DOI:** 10.1002/acn3.51214

**Published:** 2020-10-31

**Authors:** Donghong Li, Deng Luo, Junling Wang, Wei Wang, Zhangyi Yuan, Yue Xing, Jiaqing Yan, Zhiyi Sha, Horace H. Loh, Milin Zhang, Thomas R. Henry, Xiaofeng Yang

**Affiliations:** ^1^ Beijing Institute of Brain Disorders Laboratory of Brain Disorders Ministry of Science and Technology Collaborative Innovation Center for Brain Disorders Capital Medical University Beijing China; ^2^ Neuroelectrophysiological Laboratory Xuanwu Hospital Capital Medical University Beijing China; ^3^ Guangzhou Regenerative Medicine and Health Guangdong Laboratory Guangzhou China; ^4^ Department of Electronic Engineering Institute of Microelectronics Tsinghua University Beijing China; ^5^ College of Electrical and Control Engineering North China University of Technology Beijing China; ^6^ Department of Neurology University of Minnesota Minnesota USA; ^7^ Center for Magnetic Resonance Research University of Minnesota Minnesota USA

## Abstract

**Objective:**

Neuromodulatory anterior thalamic deep brain stimulation (DBS) is an effective therapy for intractable epilepsy, but few patients achieve complete seizure control with thalamic DBS. Other stimulation sites may be considered for anti‐seizure DBS. We investigated bilateral low‐frequency stimulation of the endopiriform nuclei (LFS‐EPN) to control seizures induced by intracortically implanted cobalt wire in rats.

**Methods:**

Chronic epilepsy was induced by cobalt wire implantation in the motor cortex unilaterally. Bipolar‐stimulating electrodes were implanted into the EPN bilaterally. Continuous electroencephalography (EEG) was recorded using electrodes placed into bilateral motor cortex and hippocampus CA1 areas. Spontaneous seizures were monitored by long‐term video‐EEG, and behavioral seizures were classified based on the Racine scale. Continuous 1‐Hz LFS‐EPN began on the third day after electrode implantation and was controlled by a multi‐channel stimulator. Stimulation continued until the rats had no seizures for three consecutive days.

**Results:**

Compared with the control and sham stimulation groups, the LFS‐EPN group experienced significantly fewer seizures per day and the mean Racine score of seizures was lower due to fewer generalized seizures. Ictal discharges at the epileptogenic site had significantly reduced theta band power in the LFS‐EPN group compared to the other groups.

**Interpretation:**

Bilateral LFS‐EPN attenuates cobalt wire‐induced seizures in rats by modulating epileptic networks. Reduced ictal theta power of the EEG broadband spectrum at the lesion site may be associated with the anti‐epileptogenic mechanism of LFS‐EPN. Bilateral EPN DBS may have therapeutic applications in human partial epilepsies.

## Introduction

Deep brain stimulation (DBS) targeting the anterior thalamus and other specific brain areas has been demonstrated to be effective in reducing seizures in clinical trials and in animal studies.^1^, ^2^ Neuromodulatory DBS involves the intracerebral implantation of stimulating electrodes that are connected to a subcutaneous pulse generator, which often is programmed to continuously deliver electrical pulses (“open‐loop” paradigm), or may be programmed for stimulation triggered by detection of an EEG or other type of event (“closed‐loop” paradigm). DBS of the anterior nucleus of the thalamus has been proven to be an efficacious treatment for intractable partial epilepsy and was approved for this purpose by the FDA in 2018.^3^, ^4^ The anterior nucleus of the thalamus was selected as a target for epilepsy treatment because it has dense projections to the hippocampus and widespread cortical areas; therefore, it may be an important site for modulating epileptic networks.^5^

The endopiriform nucleus (EPN) is a well‐defined nucleus of the rodent olfactory system, which contains a large, elongated concentration of multipolar neurons located deep to the piriform cortex (PC) over its full rostral to caudal extent. There are dense plexus of intrinsic connections within the EPN and dense reciprocal connections between the EPN and the overlying PC.^6^ The EPN is reciprocally connected with the amygdala, other subcortical nuclei, insular, motor, perirhinal, and entorhinal cortices.^7^, ^8^, ^9^, ^10^ Although normal functions of the EPN have yet to be fully elucidated in rodents, it has been recognized that the PC‐EPN complex can readily be targeted to initiate and propagate seizures.^11^ It has been demonstrated that the epileptiform excitatory postsynaptic potentials induced by stimulation of the in PC are driven by neurons in the subjacent EPN.^12^ Subsequent studies further showed that EPN neurons participate in and maybe injured during epileptic neural activities.^13^, ^14^ Yoshimura et al reported that lithium pilocarpine‐induced status epilepticus in rats consistently results in neuronal injury in the thalamus, amygdala, paralimbic cortices (entorhinal and piriform), and dorsal EPN,^15^ which suggests a possible role in epileptogenesis as many of these regions are similar to areas damaged in patients with temporal lobe epilepsy. Several studies have demonstrated that stimulation of the central PC interferes with amygdaloid kindling seizure.^16^, ^17^ Moreover, Bayat et al. reported that two weeks of electrical stimulation of the anterior PC reduced spontaneous seizure frequency and severity in the kainic acid‐induced spontaneous seizure model with long‐lasting effects.^18^ The EPN and PC likely act together in seizure antagonism (or promotion). Therefore, we hypothesized that the EPN may be an effective target of DBS for controlling epilepsy.

Stimulus parameters, particularly frequency, have an important impact on the effects of targeted brain stimulation for epilepsy.^19^ Mechanisms underlying frequency‐dependent effects of brain stimulation are unclear, and the parameter selection process remains largely empirical.^20^ High‐frequency stimulation (HFS) at >100 Hz may act via a mechanism causing electroencephalographic (EEG) desynchronization,^21^ whereas long‐term depression (LTD) or neuronal firing activity modulation has been suggested as the mechanism of low‐frequency stimulation (LFS, < 5 Hz).^22^, ^23^ The total energy required for LFS is relatively low during the long‐term stimulation, which may generate a lower risk of tissue injury and permit a longer battery life, compared with HFS at similar tissue current densities and pulse widths. Thus, LFS may be more advantageous than HFS in the treatment of uncontrolled epilepsy.^24^, ^25^ Several brain sites with antiepileptic responses to LFS have been identified in clinical studies ^26^, ^27^, ^28^ and animal experiments.^29^, ^30^, ^31^ However, LFS of some brain structures may result in no effect or even in the aggravation of seizures.^32^ Anti‐seizure effects of DBS are dependent on the brain region targeted and stimulation parameters.

In this study, we applied continuous low‐frequency (1Hz) electrical stimulation to the bilateral EPN of rats with cobalt wire‐induced chronic focal epilepsy. A smartphone algorithm was designed to allow for wireless control of a micro‐neurostimulator. We tested the hypothesis that bilateral low‐frequency stimulation of the endopiriform nuclei (LFS‐EPN) can reduce seizures and antagonize epileptogenesis in symptomatic partial epilepsy.

## Materials and Methods

### Animals and chemicals

Adult male Sprague–Dawley rats weighing 250‐350 g were used in this experiment. All animals were maintained in individual cages with a 12‐hour light/dark cycle with free access to water and food. We used a protocol approved by the Capital Medical University Animal Experimentation Committee. Cobalt wire was purchased from Aldrich Chemical Company, Inc. (Milwaukee WI, USA). Isoflurane was purchased from RWD Life Science Co. (Shenzhen, China), and all other chemicals were purchased from Sigma‐Aldrich (St Louis, MO, USA).

### Electrode implantation and epilepsy model

In this study, the animals were divided into three groups: the control group (n = 5) which did not have stimulation electrodes, the sham stimulation group (n = 5) which had implanted EPN electrodes that were not stimulated except for initial brief hippocampal evoked potential (EP) measurements, and the LFS‐EPN group (n = 5) which received chronic LFS of bilateral EPN depth electrodes.

**Figure 1:**
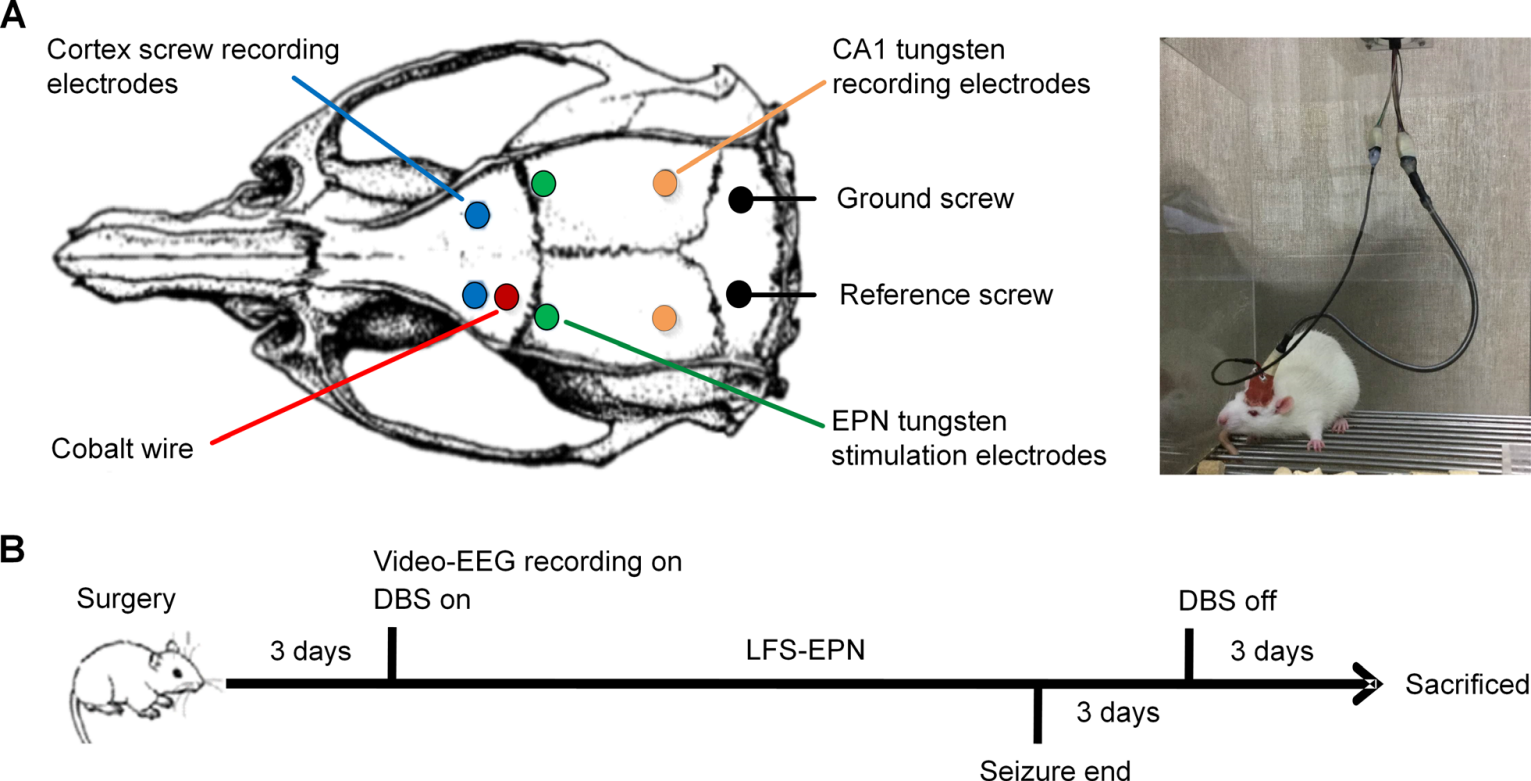
Surgical schematic diagram and experimental time flow. (A) Schematic diagram of cobalt wire and electrodes placement. (B) Experimental time flow design.

Rats were anesthetized with isoflurane (5% anesthesia induction) for 2 minutes and then anesthesia was maintained at isoflurane concentrations of 2%. After anesthesia, the rats were mounted in a stereotaxic apparatus (Stoelting, Wood Dale, IL, USA). According to the identified brain coordinates in the rat stereotaxic atlas of *Paxinos and Watson*,^33^ burr holes first were drilled through the skull. A piece of cobalt wire, 1.0 mm in diameter and 1.5 mm in length, was implanted with selected stereotaxic coordinates in the left motor cortex (AP + 2.0 mm, ML + 2.5 mm). For EEG recording, two skull screw electrodes (1.0 mm in diameter) were advanced into bilateral motor cortex (AP + 3.0 mm, ML ± 2.5 mm), then tungsten wire depth electrodes (177 μm in diameter) were implanted in the hippocampus CA1 region on each side (AP − 5.2 mm, ML ± 4.5 mm, DV −3.4 mm). In addition, reference and ground skull screw electrodes were placed extra‐axially overlying the cerebellum. For sham and stimulation group animals, custom‐made bipolar tungsten‐stimulating electrodes (diameter 177 μm, HFV‐Natural, California Fine Wire, Grover Beach, CA, USA) were stereotactically implanted into the EPN on each side (AP + 0.7 mm, ML ± 4.5 mm, DV − 7.4 ~ 8.5 mm) (Fig. 1). To confirm that each EPN stimulation electrode was implanted precisely into the EPN, we recorded the EPs generated in the hippocampus CA1 area when the ipsilateral EPN was stimulated. The EPs were elicited by trains of constant current stimuli (1 Hz, 1.0 mA, 100 μs pulse width), which were applied at increasing amplitudes across a paired cathode and anode on each side independently. An input–output curve was established for the amplitude of the EPs, from which half‐maximal stimulation intensity was determined. The half‐maximal stimulation intensity was then used for subsequent experimentation to evaluate stimulation effects on seizures in the stimulation group. All electrodes were secured with dental cement. The site of implantation of the stimulating electrode was finally confirmed by MRI and histological examination.

### The multichannel wireless neural stimulator

**Figure 2:**
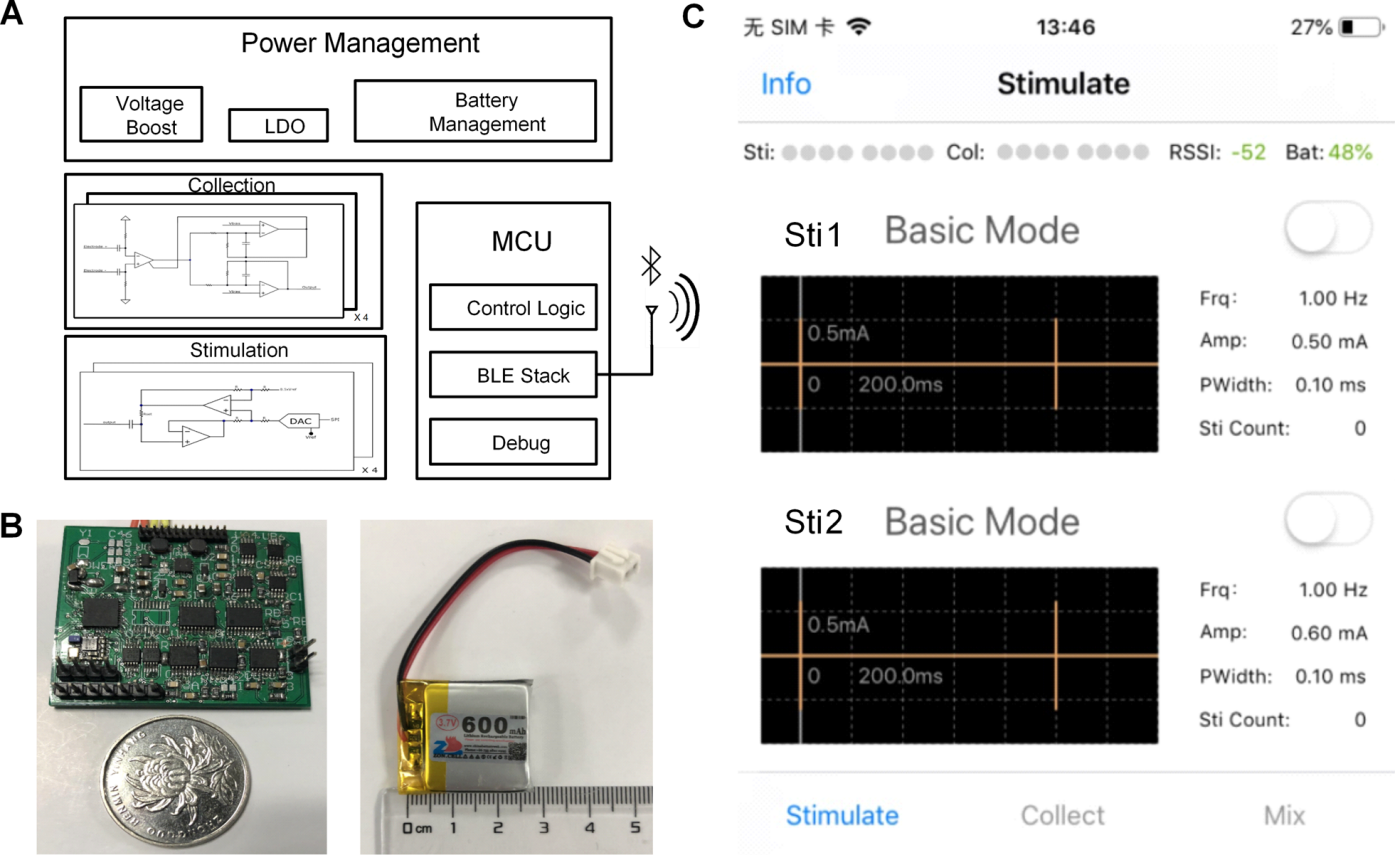
The multiple channel microneural stimulator. (A) Block diagram of the 32‐channel stimulator. (B) Configuration of the stimulator (left) and the rechargeable battery (right). (C) GUI on the smartphone. We chose the “basic mode” in our study with biphasic square‐wave pulses, 1 Hz, 100 μs per pulse, 0.5 mA, or 0.6 mA.

We developed a microneural stimulator that consists of four main functional modules: 1) a microcontroller unit (MCU) for stimulation parameter control, 2) a power management module, 3) a current stimulation generator, and 4) a stimulation output module. The design of the stimulator has been fully described in a previous paper.^34^ Fig. 2 summarizes the design of the stimulator. The stimulator can be controlled by a smartphone through a Bluetooth connection. The stimulator is portable and weighs 7.7 g with a size of 35 × 25 × 10 mm. A rechargeable battery is used to power the stimulator. An Instrumentation Operations Station (iOS)‐based Graphical User Interface (GUI) was designed for remote configuration and monitoring.

### Video‐EEG recording and electrical stimulation

Three days after surgery, all rats were put in custom‐made transparent cages and allowed to move freely. Then continuous video‐EEG monitoring was initiated and continued for the next 3 weeks. The EEG signals were recorded and digitized using a PowerLab8/35 system (ADInstruments, Colorado Springs, CO, USA) at a sampling rate of 2 kHz with synchronized video recording. Rats in the stimulation group received continuous electrical stimulation (biphasic square‐wave pulses, 1 Hz, half‐maximal stimulation intensity, 100 μs per pulse) to bilateral EPN, across a paired cathode and anode on each side, using our custom‐made neurostimulator until the rat had no seizures for three consecutive days. The rats in the sham group received no stimulation following brief measurement of EPN‐stimulation‐triggered hippocampal EPs. All experimental data were stored in a personal computer and analyzed offline using the LabChart8 software and MATLAB (Math Works, Natick, MA, USA).

### Data analysis

Seizure onset and termination were readily recognizable as abrupt changes in EEG frequency and amplitude.^35^ Distinct seizure events were defined by separation from each other by intervals of at least 10 seconds. Behavioral classification of epileptic seizures was determined by two reviewers (D.L. and Ju.W.) in a blind manner (without knowledge of the group assignment of the rat). The seizure scale criteria based on the Racine scale were as follows; 1: staring and mouth clonus; 2: head nodding; 3: unilateral forelimb clonus; 4: rearing and bilateral forelimb clonus; 5: rearing and falling.^36^ Stages 1‐3 represented partial forebrain seizures and stages 4‐5 seizures were generalized forebrain seizures.^37^

To explore the effect of LFS‐EPN on EEG power at the epileptogenic zone during seizures, Fast Fourier Transformation (FFT) was used to calculate the power spectral density (PSD) of the standard frequency band during seizures. We calculated the PSD value in 7 frequency bands: delta (1‐4 Hz), theta (4‐8 Hz), alpha (8‐13 Hz), beta (13‐30 Hz), gamma (30‐80 Hz), ripples (80‐200 Hz), and fast ripples (200‐500 Hz). We observed large individual variability in EEG power between individual rats; therefore, we normalized the PSD value of EEG power in different frequency bands for each rat. We randomly chose three non‐seizure EEG segments with a duration approximately equal to the mean seizure duration as baseline for each rat in each group. Then we calculated and compared the ictal‐to‐interictal ratio of EEG PSD value in different frequency bands between seizures and baseline between groups.

### Magnetic resonance imaging

When video‐EEG recording was finished, rats were euthanized with an overdose of isoflurane and then perfused transcardially with saline and 4% paraformaldehyde in 0.1 M phosphate buffer. Then the brain was removed immediately after the perfusion and post‐fixed in 4% phosphate‐buffered paraformaldehyde at 4 °C overnight. After removal of the ferromagnetic cobalt wire, brains of rats from the LFS‐EPN group were imaged using standard magnetic resonance techniques. The fixed brains were placed in a custom‐built magnetic resonance imaging (MRI)‐compatible tube and scanned on a small animal 7T MRI unit, the BioSpec 70/20 USR (PharmaScan 7T, Bruker, Germany). Anatomical images for each rat were acquired in the coronal plane using the RARE sequence with the following parameters: repetition time (TR), 3200 ms; echo time (TE), 30 ms; field of view (FOV), 28 × 28 mm; matrix size, 256 × 256; in‐plane resolution, 109 × 109 μm; slice thickness, 500 μm; and no gap. The anterior–posterior extent of MRI signal for rats were acquired using the RARE sequence with the following parameters: repetition time (TR), 3000 ms; echo time (TE), 45 ms; field of view (FOV), 32 × 32 mm; 256 × 256 matrix size; in‐plane resolution, 109 × 109 μm; and slice thickness, 1000 μm, no gap.

### Histology

After the magnetic resonance imaging data were acquired, the brains were equilibrated in 30% (w/v) sucrose solution. Immunohistochemistry was used to confirm the accurate placement of stimulation electrodes and to observe whether the stimulation process caused any damage to surrounding tissues. Frozen brain sections were made using a Cryostat Microtome (Leica, Germany). The brain sections were then stained using Nissl and GFAP staining to confirm the placement site of the stimulating electrode and evaluate for tissue injury. Only those rats with accurate placement of stimulating electrodes were used for data analysis.

### Statistical analysis

All experimental data were analyzed using SPSS software 22.0 (IBM, Armonk, New York, USA) and presented as the mean ± SEM. Statistical analysis was performed using two‐tailed unpaired t‐tests or repeated measures one‐way ANOVA followed by Least Significant Difference *post hoc* test. A P‐value of less than 0.05 was considered statistically significant.

## Results

### Evoked potentials

**Figure 3:**
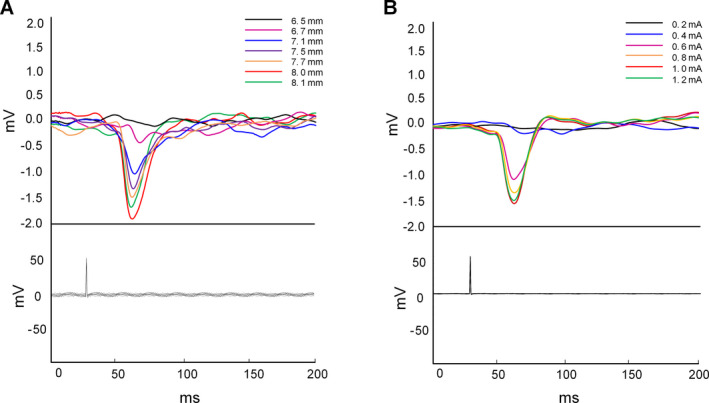
Evoked potentials recorded from hippocampal upon ipsilateral EPN stimulation. (A) The EP was applied to confirm the location of electrode implantation. As the stimulus electrode approached the EPN, the EP amplitude increases gradually. When the EP amplitude reached the maximum, the stimulus electrode is implanted into EPN accurately. (B) Determination of experimental stimulus intensity. Once the location of the EPN was determined, we increased the stimulation current from 0.2 mA until the maximum EPs appeared. Then the half‐maximal stimulation strength was used in subsequent chronic stimulation experiments.

Stimulation of the EPN produced parameter‐dependent effects on hippocampal activity. As is shown in Fig. 3, robust, short‐latency EPs were recorded in the hippocampal CA1 region in response to EPN stimulation, and the valley point of these responses had a latency of approximately 30‐35 ms from stimulus onset. These EPs demonstrated a classic input‐output relationship between the EPN and hippocampus, which suggests increasing recruitment of the underlying neural network. In our experiments, among all the subjects, the current intensity to induce the maximum EPs was 1.0 mA or 1.2 mA. Thus, the current intensity used in the LFS‐EPN group was half of the maximum current and ranged from 500 μA to 600 μA.

### Cobalt wire‐induced chronic epilepsy in the control group

**Figure 4:**
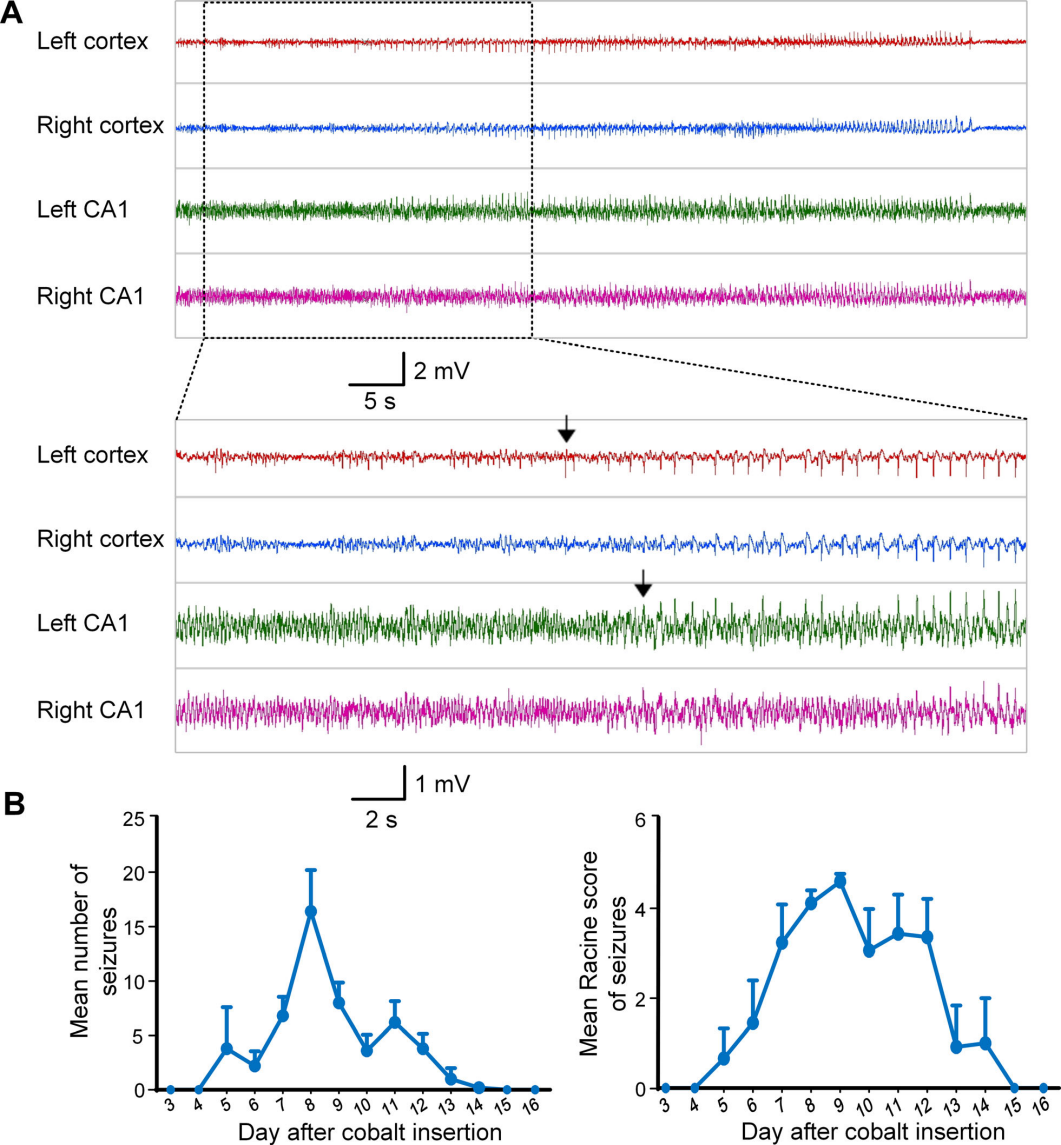
Cobalt wire induced chronic epilepsy in control group. (A) Example of seizure which corresponding seizure was stage 5 on the Racine scale. The black arrows indicate the onset of the seizure. The EEG indicated that the electrical seizures first started in the left motor cortex (cobalt wire implanted region). (B) Mean number and mean Racine score of spontaneous seizures plotted by time after cobalt wire insertion. Data were represented as mean ± SEM (n = 5).

All rats implanted with cobalt wire developed spontaneous seizures after 6 to 8 days, and the seizures lasted from 5 to 8 days. (Fig. 4). As expected, the electrographic seizures first occurred in the left motor cortex before propagation to other sites, consistent with cobalt wire‐induced chronic epilepsy as a neocortical focal epilepsy model. The mean number of seizures per day in the control group was 8.1 ± 1.0/d and the total number of seizures was 52.0 ± 4.3. The average Racine score for individual seizures was 4.1 ± 0.1, and the average total Racine score for each animal combining all seizure activity was 212.0 ± 13.6. At the ictal onset zone in the control group, the mean duration of seizures was 56.1 ± 11.7 seconds, and the total duration of seizures was 2942.2 ± 683.7 seconds.

### Stimulating electrode implantation did not alter seizure occurrence and properties

We analyzed and compared the latency, Racine score, number, and duration of cobalt wire‐induced seizures between the control and the sham stimulation groups. We found no statistically significant difference in the latency and the mean number of seizures between the sham stimulation group (5.4 ± 0.2 days, 7.6 ± 0.8 per day) and control group (6.6 ± 0.5 days, 8.1 ± 1.0 per day), *P* = 0.06 and *P* = 0.59, respectively. In addition, on comparing the mean Racine score and the mean duration of seizures between the two groups, we also found no statistical difference (*P* > 0.05). These results indicate that the implantation of stimulating electrodes did not significantly alter the occurrence and properties of seizures.

### Effects of LFS‐EPN on spontaneous seizures in the chronic epilepsy rat models

**Figure 5:**
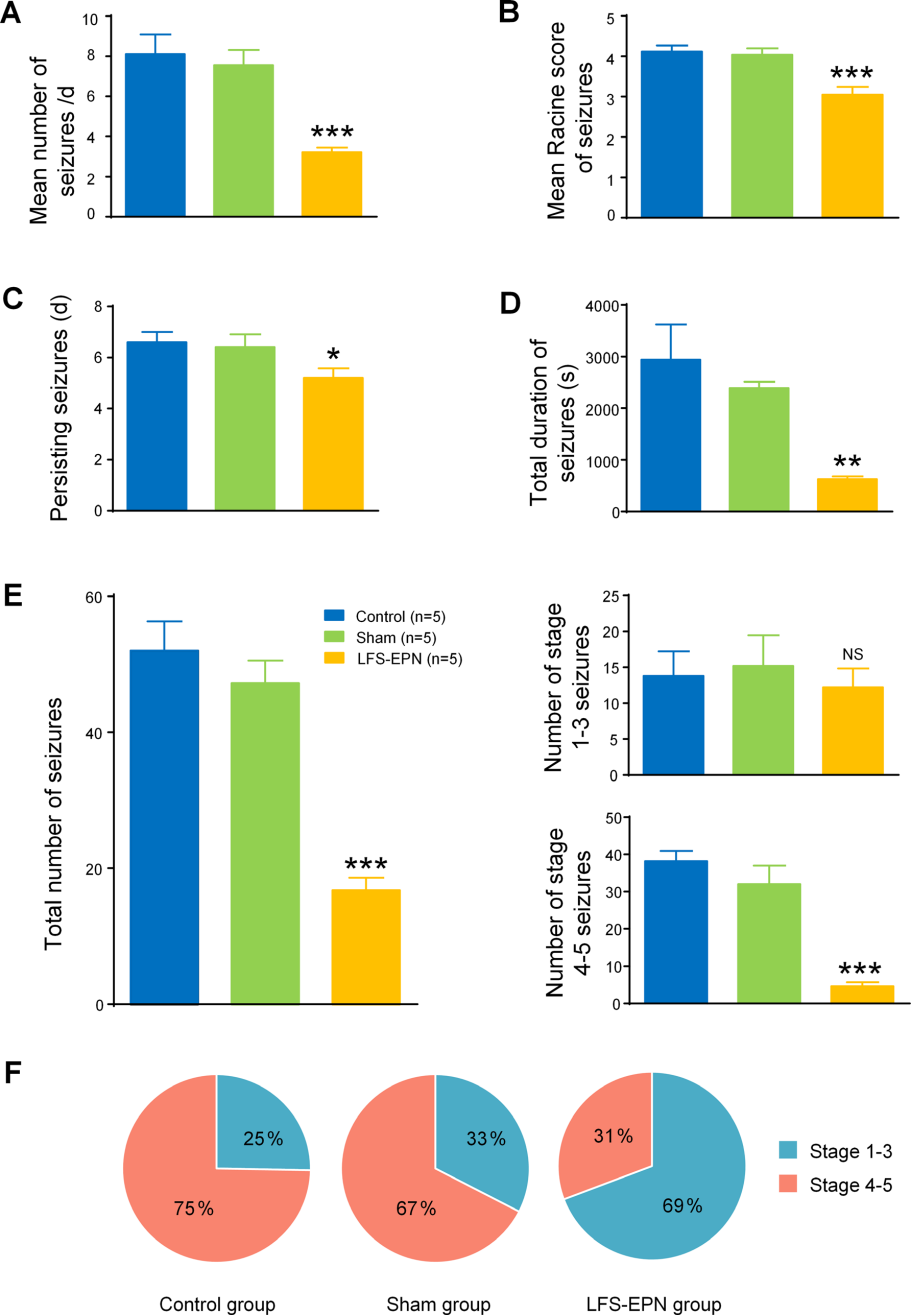
Effects of LFS‐EPN on spontaneous seizures in the rat chronic epileptic model. (A) LFS‐EPN significantly reduced the frequency of seizures. (B) LFS‐EPN lowered the mean Racine score of seizures. (C) LFS‐EPN significantly reduced persisting seizures. (D) There was a significant reduction in the total seizure duration in the LFS‐EPN group. (E) Although LFS‐EPN significantly reduced the total number of seizures (left), however, it only significantly reduced the number of stage 4‐5 seizures (right, bottom histogram), but did not reduce the number of stage 1‐3 seizures (right, top histogram) (Mean ± SEM, *P < 0.05, **P < 0.01, ***P < 0.001). (F) Data are represented as percentage of number of seizures with 1‐3 stage (blue slice), 4‐5 stage (red slice) seizures in the control group (left pie chart), sham stimulation group (middle pie chart), and LFS‐EPN group (right pie chart).

In this study, bilateral LFS‐EPN significantly decreased the mean frequency of seizures to 3.2 ± 0.2 per day compared to 8.1 ± 1.0 per day in the control group (*P* < 0.001) and 7.6 ± 0.8 per day in the sham stimulation group (*P* < 0.001) (Fig. 5A). It also reduced the Racine score over the period of seizure occurrence in the EPN stimulation group (3.0 ± 0.2) compared to the control group (4.1 ± 0.1, *P* < 0.001) and the sham stimulation group (4.0 ± 0.2, *P* < 0.001) (Fig. 5B). In addition, the number of days the seizure persisted in the LFS‐EPN group was reduced to 5.2 ± 0.4 compared to 6.6 ± 0.4 days in the control group (*P* < 0.05) and 6.4 ± 0.5 days in the sham stimulation group (*P* < 0.05) (Fig. 5C). The total seizure duration in the LFS‐EPN group was 628.0 ± 53.7 seconds, which was significantly shorter than 2942.2 ± 683.7 s in the control group and 2390.4 ± 119.9 s in the sham stimulation group (*P* < 0.01) (Fig. 5D). Also, LFS‐EPN reduced the total number of seizures to 16.8 ± 1.8 compared 52.0 ± 4.3 in the control group and 47.2 ± 3.4 in the sham stimulation group (*P* < 0.001) (Fig. 5E). Compared with the control group and the sham stimulation group, LFS‐EPN significantly reduced the number of stage 4‐5 seizures (*P* < 0.001), but did not reduce the number of 1‐3 stage seizures (*P*> 0.05). The majority of seizures in the control group (75%) and the sham group (67%) were stage 4‐5 generalized forebrain seizures. In the stimulation group, only 31% of the rats had stage 4‐5 seizures (*P* < 0.01, Fig. 5F).

### The effects of LFS‐EPN on seizures recorded in different brain regions

**Figure 6:**
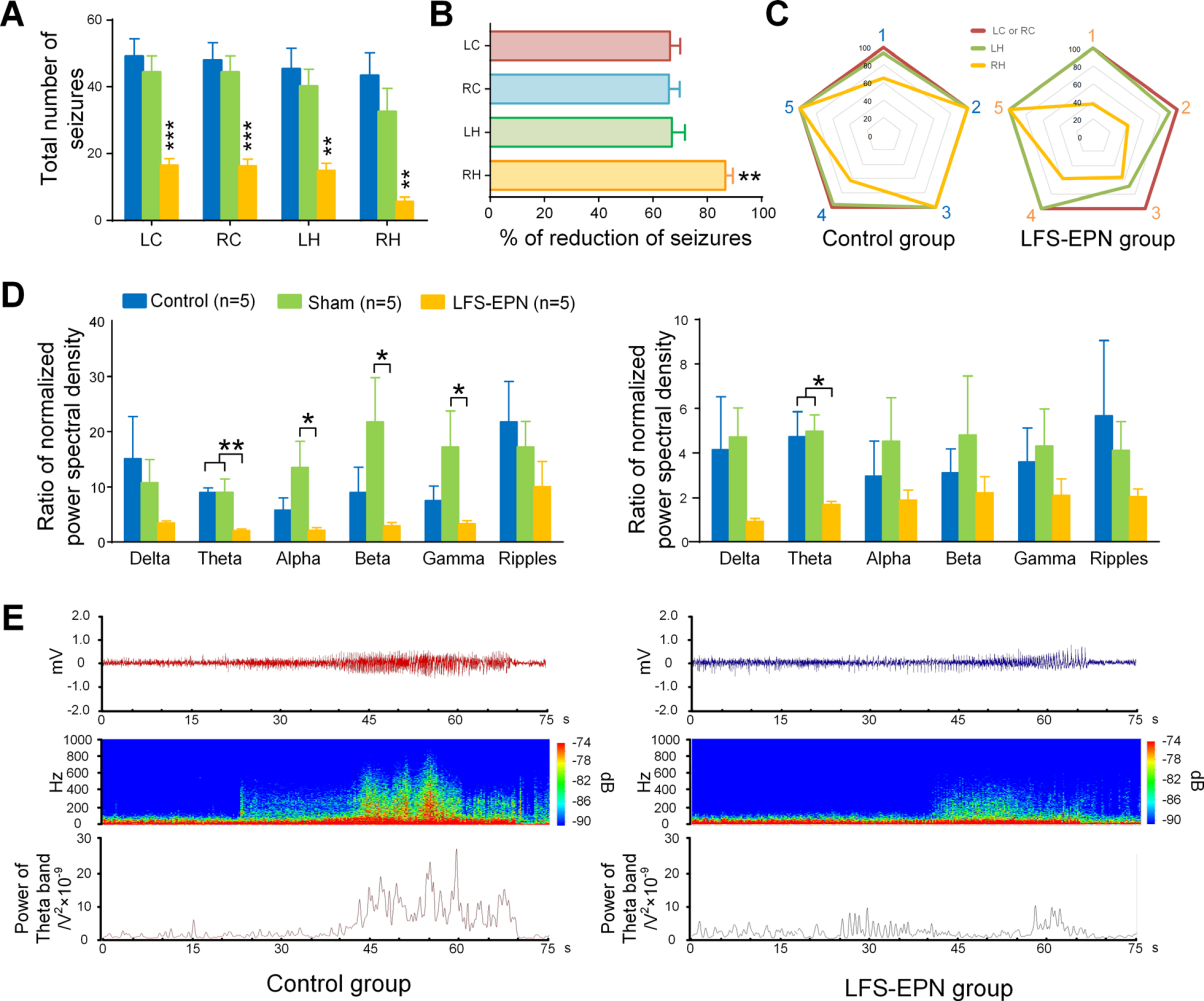
The effects of LFS‐EPN on seizures recorded in different brain regions. (A) LFS‐EPN significantly decreased the total number of seizures in the left cortex (LC), right cortex (RC), left hippocampal (LH) and right hippocampal (RH) compared with the control group and sham stimulation group. (B) The total number of seizures was mostly reduced in RH compared with LC, RC, and LH (P < 0.01). (C) The radar chart shows the detailed percentage of four different regions between the control group and LFS‐EPN group with five subjects in each group. Number 1‐5 in the chart represented the code number of the rats within groups. (D) The comparison of normalized power spectra intensity of the EEG frequency spectrum of cobalt wire‐induced seizures in cortex (left histogram) and hippocampal (right histogram). Data are displayed as mean ± SEM, one‐way ANOVA followed by LSD *post hoc* test, * P < 0.05, ** P < 0.01, *** P < 0.001. (E) Representative examples of EEG (top) derived from the control and LFS‐EPN group, as well as the total power of the EEG (middle), and the power of theta band displayed with curve (bottom). The EEG were selected from the left CA1 on the 8th day after cobalt wire insertion.

Considering that the EPN is widely connected with parahippocampal and subcortical areas, we were interested to test whether LFS‐EPN has a modulating effect on large‐scale epileptic networks. To prove it, we analyzed and compared the total number of seizures in four different regions, including the left cortex (epileptic focus), right cortex (contralateral to the epileptic focus), left hippocampus (ipsilateral but remote from the epileptic focus) and right hippocampus (contralateral to and remote from epileptic focus). Our results showed that compared to the control group and sham stimulation group, all the total number of seizures recorded from the above four different regions were significantly reduced following LFS‐EPN (*P* < 0.01, Fig. 6A). The right hippocampus showed an 86.6% reduction in the number of seizures, which was a significantly greater seizure reduction compared to the other three regions (with reductions of 66.3% in the left cortex, 65.8% in the right cortex, and 67.0% in the left hippocampus) at *P* < 0.01 for each comparison (see Fig. 6B‐6C). After calculating the ratio of EEG PSD in different frequency bands between seizures and baseline, we found that, in the cortex, the ratio of PSD in theta bands was significantly reduced in the LFS‐EPN group (2.1 ± 0.3) compared to the control (9.0 ± 0.9) and sham groups (9.0 ± 2.4) (*P* < 0.05, Fig. 6D). In the hippocampal CA1 region, the same ratio was reduced from 4.7 ± 1.1 (control group) and 5.0 ± 0.7 (sham group) to 1.7 ± 0.1 (LFS‐EPN group) (*P* < 0.05, Fig. 6D, right). In addition, there were no statistical differences between the groups in the ratio of PSD in alpha, beta, and gamma bands.

### Magnetic resonance imaging

**Figure 7:**
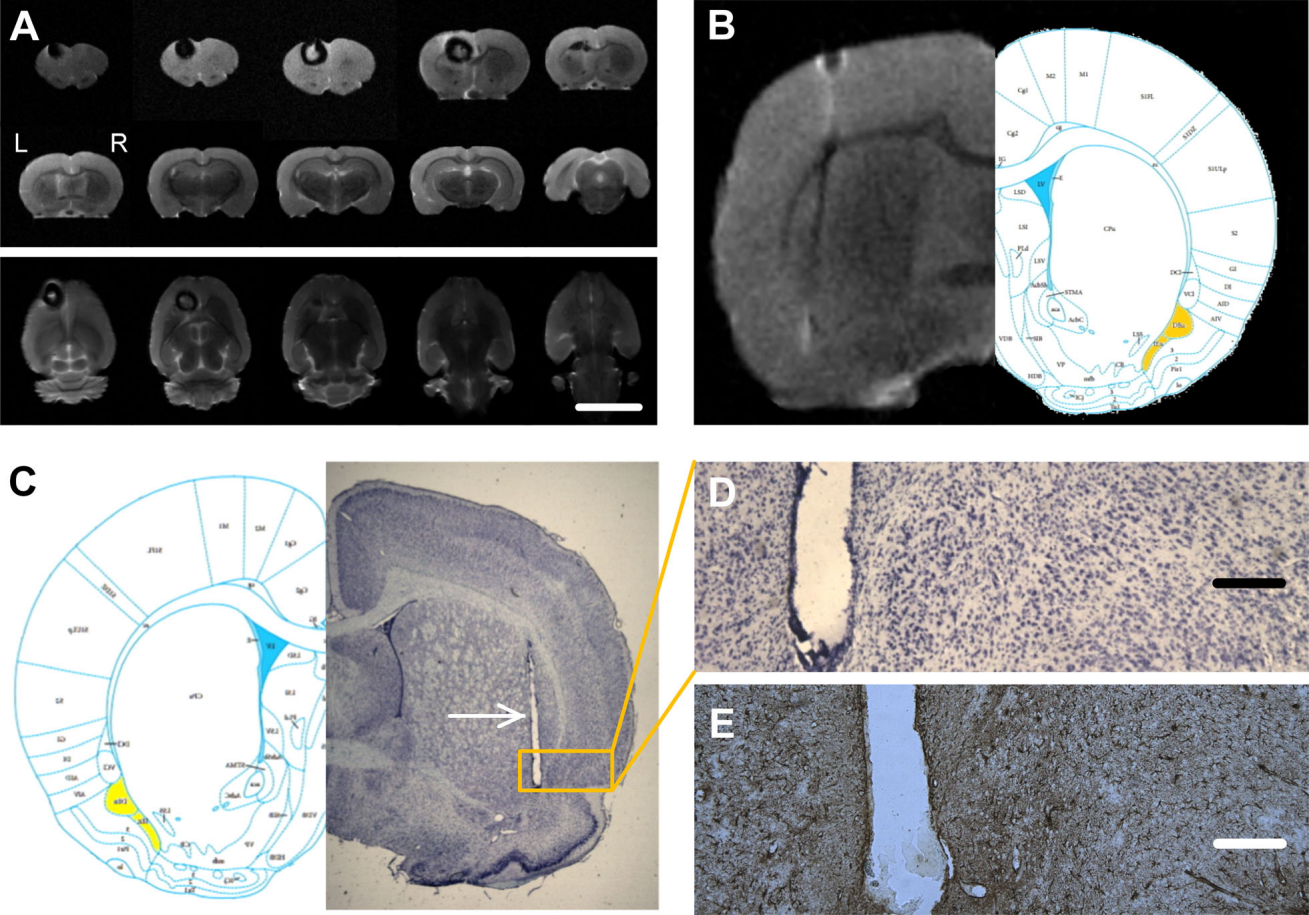
The location of the stimulating electrodes and epileptogenic areas were identified by MRI technique and Nissl’s staining. (A) Representative coronal images collected from fixed brain after removal of the cobalt wire (top) show MRI signal loss (dark) in the left frontal cortex and axial images of MRI signal loss (bottom). Scale 1 cm for both images. (B) The MR images of the EPN sites location are well merged with the rat brain atlas. (C) Location of EPN in the Nissl’ staining. A unilateral brain slice with the electrode placement in the EPN is shown, opposite the corresponding section from a rat atlas with the target brain area outlined in yellow. The rupture shows the line direction of the stimulating electrode (white arrow). (D) Higher magnifications of area indicated in left (yellow box) by Nissl’ staining shows there is no significant neuronal death. (E) GFAP staining shows there is no significant glial cell proliferation around the implanted electrodes. Calibrations in A is 1 cm and in D and E are 250 µm.

We utilized MRI imaging of fixed brains to confirm accurate localization of the EPN‐stimulating electrodes in the brain tissue. The location of all EPN electrodes based on MRI was anatomically accurate based on a rat brain atlas. There was a loss of MRI signal surrounding the implanted ferromagnetic cobalt wire, and the appearance of this artifact was consistent with the presence of cobalt in tissue both in coronal and axial images (Fig. 7A to B).

### Histological results

The brains of all rats underwent histological examination. We used Nissl staining combined with MRI to confirm whether the stimulation electrode was implanted in the accurate location (Fig. 7C). Only those rats with accurate placement of the EPN electrodes were used in data analysis. Three rats were excluded from the analysis because of inaccurate electrode placement. In addition, Nissl and GFAP staining revealed there was no detectable neuronal death or glial cell proliferation around the implanted electrodes in all stimulation and sham group rats (Fig. 7D to E).

## Discussion

We found that sustained 1‐Hz LFS of bilateral EPN significantly attenuated cobalt wire‐induced chronic epilepsy in rats. Bilateral LFS‐EPN significantly reduced daily frequency of electrical seizures, rates of full seizure generalization, and seizure duration. These results indicate that LFS‐EPN not only can reduce seizures in active epilepsy but can also antagonize epileptogenesis in this model, suggesting that EPN may be a promising target for therapy in human epilepsy. This is the first study using low‐frequency stimulation of EPN to control seizures by modulating the epilepsy network in an animal model.

Epilepsy is increasingly understood as a network disorder,^38^ with seizure‐generating “foci” embedded in webs of structural and functional connections.^39^ Many researches have shown that EPN may be involved in the propagation of seizures under the condition of altered excitability during the epileptic process.^40^ The PC has been demonstrated to be important in the seizure propagation network^41^ and previous studies have demonstrated that LFS of the PC can delay seizure development in epilepsy models.^16^ In humans, the EPN is situated farther from major blood vessels than is the PC, possibly making the EPN a safer location for electrode implantation. Therefore, we selected EPN as a new target of DBS for epilepsy in this study.

It is generally accepted that stimulus site and frequency determine the effects of DBS for epilepsy.^19^, ^20^, ^21^, ^22^, ^23^, ^47^ However, the mechanisms underlying frequency‐dependent effects of brain stimulation are unclear. Clinical studies have suggested that the possible mechanism of LFS includes the induction of LTD and activation of GABA‐benzodiazepine and endogenous opioid systems.^48^ A LFS study in rats indicated that actions on GABA‐benzodiazepine and endogenous opioid systems decrease neuronal excitability.^30^ Other studies showed that LFS could suppress the after‐discharge duration of seizures,^23^, ^31^ which suggested that the discharge threshold was raised following long‐term LFS. Our results showed that LFS‐EPN can significantly reduce generalization of seizures in Racine stages 4‐5 in the accepted interpretation of the Racine scale in rodents.^49^ Several studies using optogenetics and extracellular electrophysiological recording in mouse models have found that activation of GABAergic neurons retarded generalization by inhibiting the firing of pyramidal neuron,^50^, ^51^ suggesting that LFS at an early stage may be more effective in antagonizing epileptogenesis.^52^

Altered dynamics of the stimulated network, recalibrating dysfunctional circuits, maybe a core mechanism of DBS.^53^ Our results showed that the effect of LFS‐EPN on hippocampus seizure control was better than that of motor cortex, which may be due to denser EPN connectivity with entorhinal cortex,^6^, ^40^ resulting in greater modulation of the temporal lobe than the motor cortex.^54^, ^55^ EPN may be involved in the propagation and generalization of seizures originating from the motor cortex to the hippocampus. Anatomical evidence does not demonstrate efferent projections from EPN to the structures in the contralateral hemisphere of the brain.^56^, ^57^ In addition, given that seizures in cobalt‐induced chronic epilepsy originate in unilateral motor cortex and then propagate to hippocampus, and that LFS‐EPN has a stronger inhibitory effect on ictal discharges in the hippocampus than in the motor cortex, we suspect that bilateral LFS‐EPN limited the propagation of seizures by regulating the epileptic network.

We found that LFS‐EPN lowered the power of the theta band at the ictal onset zone, with relatively less of an effect on other frequency bands. Specifically, on comparing frequencies of ictal discharges in the motor cortex across the three groups, we found that theta power was significantly lower in the LFS‐EPN group versus the other groups, with no significant differences of ictal discharge power in other frequency bands. The suppression of the ictal motor cortex theta‐band oscillations by remote stimulation of EPN adds further evidence regarding potential mechanisms by which LFS‐EPN can reduce seizure activity. There has been a significant amount of research pertaining to the relationship between theta waves and the generation of seizures. Stypulkowski et al. reported that DBS of non‐epileptic animals induced an inhibition in the theta band power of local field potential (LFP).^58^ A previous study in pilocarpine‐induced epilepsy rats reported that much of the increased preictal firing of neurons in the subiculum and CA1 correlated with preictal theta activity.^59^ Furthermore, studies of rapid focal cooling^60^ and photolysis of cage‐GABA^61^ in controlling seizures also demonstrated that theta band power reduction is a mechanistic marker for seizure reduction in these models. Several studies in other epilepsy models have found enhanced theta oscillations to be associated with reduced seizures^62^, ^63^; however, the correlation between the change in theta‐band power and epileptic seizures needs to be further clarified.

The translation of our findings to human therapy will be a laborious endeavor, but one that is already supported by findings from earlier clinical investigations. Reports in patients with partial epilepsy suggest mechanistic relevance of the EPN structures in partial epilepsy networks.^64^, ^65^, ^66^ Perhaps PC and EPN stimulation may be similar in their effects in human epilepsy, as has been demonstrated in animal models. A further consideration is the greatly altered morphology of human mesial frontotemporal limbic system structures compared with those of subprimate mammals, particularly noting the proximity of the middle cerebral artery to much of the piriform region.^67^ The PC seems an unsafe target for subdural electrode or depth electrode placement in humans, but the EPN may be more suitable for depth electrode implantation.

In conclusion, our findings indicated that LFS‐EPN can significantly reduce seizure frequency, duration, and intensity in a chronic epilepsy rat model. In addition, LFS intervention at an early stage of seizures onset may be critical for inhibiting propagation of epilepsy by regulating the epileptic network. Another important finding in our experiment is that reduced power of the theta band, maybe a key player in the mechanism by which LFS‐EPN controls seizures. This study provides a theoretical basis for future clinical translational studies using the EPN as a new target for DBS in human therapy.

## Conflicts of Interest

The authors do not have any conflict of interest.
